# Integrative multi-omics characterization of KLRB1/CD161 in diffuse large B-cell lymphoma: associations with immune context, subtype biology, and therapeutic response

**DOI:** 10.3389/fmolb.2026.1903725

**Published:** 2026-09-24

**Authors:** Erwei Li, Yingying Tang, Yuanyuan Wanyan, Yu Liang

**Affiliations:** Department of Blood Transfusion, Fuwai Central China Cardiovascular Hospital, Henan Cardiovascular Disease Center (Central China Subcenter of National Center for Cardiovascular Diseases), Central China Fuwai Hospital of Zhengzhou University, Henan Provincial Cardiovascular Hospital, Zhengzhou, China

**Keywords:** CD161, DLBCL, immune checkpoint, Klrb1, multi-omics integration, tumor immune microenvironment

## Abstract

**Background:**

The tumor immune microenvironment (TME) of diffuse large B-cell lymphoma (DLBCL) displays pronounced heterogeneity, and existing immunotherapeutic approaches achieve only suboptimal outcomes for a considerable proportion of affected individuals. CD161—a C-type lectin receptor whose coding gene is KLRB1—has lately emerged as a modulator of T-cell activity in several malignancies; nonetheless, a thorough characterization of its function in DLBCL has not yet been undertaken.

**Methods:**

Transcriptomic analyses quantified KLRB1 mRNA, whereas flow cytometry and immunoblotting assessed its encoded surface protein, CD161. Transcriptomic, genomic, epigenomic (methylation), and exploratory single-cell RNA-sequencing datasets from DLBCL cohorts were integrated with *in vitro* functional assays to characterize clinical associations, immune context, and hypothesis-generating therapeutic relationships.

**Results:**

Pan-cancer analysis revealed predominantly inverse associations between KLRB1 mRNA and immunomodulatory features, whereas DLBCL bulk-tumor analyses showed positive correlations with MHC molecules, immune-cell infiltration estimates, CD8^+^ T-cell effector programs, and immune-checkpoint transcripts. KLRB1 mRNA was higher in ABC than in GCB DLBCL and was associated with vital status at last follow-up and clinical stage. Lower TIDE scores and pharmacogenomic modeling suggested exploratory, context-dependent relationships with immune-checkpoint blockade and drug response; however, the IMvigor210 urothelial carcinoma cohort showed no significant objective-response difference. Single-cell analyses from two biopsies were exploratory. In OCI-LY3 cells, CD161 manipulation altered MHC-I, phospho-mTOR, cytokine secretion, T-cell activation, and fixed-dose drug-induced apoptosis. These two-biopsy single-cell outputs were not treated as reproducible trajectories or validated intercellular signaling networks, and all functional findings remained specific to the OCI-LY3 model.

**Conclusion:**

The KLRB1 mRNA/CD161 protein axis is associated with an immune-inflamed DLBCL phenotype and ABC subtype biology. Its prognostic and treatment-predictive value remains hypothesis-generating and requires validation in multivariable survival models, DLBCL-specific immunotherapy cohorts, additional cell lines, and *in vivo* systems. Confirmation in a GCB cell line and formal dasatinib and doxorubicin dose-response experiments are specifically required.

## Introduction

Among newly diagnosed lymphoma cases across the globe, B-cell neoplasms constitute more than 80%, with diffuse large B-cell lymphoma (DLBCL) being the commonest and most clinically aggressive non-Hodgkin lymphoma subtype (Arffman et al., 2024). The disease is characterized by profound biological diversity, giving rise to pronounced disparities in how patients present, how their disease evolves, and how they respond to treatment (Crump et al., 2017; Sun et al., 2024). Underlying this diversity is a landscape of genetic and epigenetic aberrations that drive both lymphomagenesis and clonal evolution (Pasqualucci and Dalla-Favera, 2018; Li et al., 2024), rendering accurate prognostication and therapeutic decision-making particularly challenging. On the basis of cell-of-origin transcriptional signatures, DLBCL has been subclassified into two major molecular categories: germinal center B-cell-like (GCB) and activated B-cell-like (ABC), each exhibiting differential sensitivity to standard rituximab-containing immunochemotherapy (Alizadeh et al., 2000). Although the introduction of rituximab-based combination regimens and chimeric antigen receptor T-cell (CAR-T) therapy has led to considerable gains in long-term survival (Lee et al., 2024; Salles et al., 2020), roughly one-third of individuals either fail to achieve durable remission or develop chemorefractory disease, emphasizing the pressing demand for novel biomarkers capable of improving risk-based stratification and exposing fresh therapeutic opportunities (Zhang et al., 2022; Zeng et al., 2022). A recent review similarly emphasizes that DLBCL progression and treatment response reflect interacting genetic, signaling, and immune-microenvironmental determinants (Sarode et al., 2025).

Encoded by the KLRB1 gene, CD161 is a type II transmembrane protein belonging to the C-type lectin-like family and is found on the surface of natural killer (NK) cells, NKT cells, and particular CD4^+^ and CD8^+^ T-cell subsets (Chen et al., 2024; Hu et al., 2024; Konduri et al., 2020). Although KLRB1-expressing T cells harbor a broad T-cell receptor repertoire, their functional properties closely mirror those of innate-like T lymphocytes (Mathewson et al., 2021). Of particular importance, CLEC2D (also termed LLT1)—the cognate ligand for CD161—is present on both neoplastic cells and immunosuppressive myeloid elements within the TME, implying that signaling through the CD161–CLEC2D pair may operate in a manner analogous to the PD-1–PD-L1 axis (Mathewson et al., 2021). Interference with CLEC2D–CD161 engagement, achieved through either blocking antibodies or targeted deletion of CD161, amplifies the anti-neoplastic capacity of defined T-cell populations and synergizes with PD-1 blockade across several human tumor types (Mathewson et al., 2021; Gentles et al., 2015; Braud et al., 2018). In the context of HPV-driven oropharyngeal squamous cell carcinoma, CD161 demarcates cytotoxic T lymphocyte subsets pursuing divergent differentiation trajectories, and an elevated intratumoral frequency of CD161+ CTLs has been linked to superior treatment outcomes and extended overall survival (Wei et al., 2023). Additionally, the CD161–CLEC2D/LLT1 interaction suppresses NK cell-mediated lysis of target cells (Braud et al., 2022). Notwithstanding this body of knowledge, how alterations in CD161 expression influence tumor biology and clinical behavior specifically within the complex and variable TME of DLBCL has yet to be thoroughly elucidated.

Here, we began with a pan-cancer survey of KLRB1 mRNA and immune-related parameters, followed by DLBCL-focused analyses of immune context, genomic and epigenomic features, clinicopathological associations, and computational estimates of therapeutic response. Single-cell analyses were used to explore KLRB1-associated transcriptional programs and cellular interactions, and cell-based assays examined the functional consequences of manipulating the encoded CD161 protein. Given the observational nature of the patient-level analyses and the limited experimental models, the study was designed to generate mechanistic and translational hypotheses rather than establish KLRB1/CD161 as an independent prognostic or treatment-predictive biomarker. The two-biopsy single-cell component was therefore restricted to descriptive hypothesis generation and was not used to assert reproducible lineage trajectories or active ligand-receptor signaling.

## Materials and methods

### Data acquisition and preprocessing

Transcriptomic profiles and matched clinical information for DLBCL were obtained from TCGA-DLBC. Four GEO series were used after accession-level verification: GSE4475, GSE10846, GSE32918, and GSE53786. Inter-study technical variation among GSE4475, GSE10846, and TCGA-DLBC was addressed using the ComBat method in the sva R package. GSE32918 and GSE53786 were retained as independent validation datasets. Dataset platforms, sample sizes, available clinical information, and analytic roles are summarized in Table 1.

**TABLE 1:** Summary of datasets and their roles in the study.

Dataset	Platform/data type	Sample size	Clinical information	Role in this study
TCGA-DLBC	RNA-seq; mutation, copy-number and 450K methylation data	48 cases; available sample number varied by data layer	Stage, vital status and other available clinicopathological variables	Discovery and multi-omics analyses
GSE4475	GPL96; Affymetrix HG-U133A	220 unique mature aggressive B-cell lymphoma specimens (221 arrays); DLBCL subset used	Cell of origin and available clinical follow-up	Expression/subtype validation
GSE10846	GPL570; Affymetrix HG-U133 plus 2.0	420 DLBCL samples (181 CHOP and 233 R-CHOP clinical samples reported in the series design)	Treatment and survival follow-up	Expression and clinical validation
GSE32918	GPL8432; Illumina HumanRef-8 WG-DASL v3.0	172 DLBCL patients (249 arrays including technical replicates)	Cell of origin, treatment and follow-up	Independent validation
GSE53786	GPL570; Affymetrix HG-U133 plus 2.0	119 clinical samples	Cell-of-origin classification	Independent subtype validation
heiDATA scRNA-seq cohort	Single-cell RNA sequencing	2 DLBCL lymph-node biopsies	Cell-type-resolved transcriptomes	Exploratory single-cell analysis
IMvigor210	Bulk transcriptome and anti-PD-L1 response data	Metastatic urothelial carcinoma cohort	Objective response to atezolizumab	Non-DLBCL external, exploratory comparison

### Terminology and group definition

Throughout the transcriptomic, genomic, epigenomic, and single-cell analyses, the measured variable is KLRB1 mRNA. The term CD161 is reserved for the encoded protein measured by flow cytometry or immunoblotting, or for discussion of the receptor-ligand axis.

Within each cohort, KLRB1 mRNA was retained as a continuous variable for correlation-based immune, genomic, and pathway analyses. Cohort-specific median dichotomization was used only for the prespecified group comparisons shown in the corresponding figures. Because alternative threshold sensitivity analyses were not available, all cutoff-dependent results were interpreted as exploratory rather than as evidence for a clinically transferable decision threshold.

### Evaluation of tumor immune microenvironment characteristics

We initially evaluated, on a pan-cancer scale, associations between KLRB1 mRNA abundance and immune-related features, including immunomodulator expression, CIBERSORT-derived tumor-infiltrating immune-cell estimates, and inhibitory immune-checkpoint transcripts. These bulk-tumor correlations were interpreted as composite signals that may reflect both cellular expression and variation in immune infiltration or tumor purity. The same features were then compared between the prespecified KLRB1-high and KLRB1-low DLBCL groups.

### Association with tumor mutational burden and microsatellite instability

Pan-cancer TMB and MSI metrics were obtained from the datasets used in the original analysis. Associations of KLRB1 mRNA abundance with TMB and MSI were evaluated across tumor types using Spearman rank correlation. Within DLBCL, TMB and MSI were additionally compared between the prespecified KLRB1-high and KLRB1-low groups.

### Correlation with clinical features and molecular subtypes

Associations of KLRB1 mRNA abundance with clinical variables, including vital status at last follow-up, disease stage, sex, and age, were examined. KLRB1 mRNA was compared among ABC, GCB, and unclassified DLBCL subtypes, with subtype-related expression patterns evaluated in GSE4475 and GSE10846. Because vital-status comparisons are not equivalent to time-to-event analyses, these analyses were treated as clinical association analyses rather than proof of independent prognostic value.

### Pathway enrichment analysis

Enrichment scores for immunotherapy-response gene signatures, TME-associated gene sets, and the MSigDB Hallmark collection were calculated by single-sample Gene Set Enrichment Analysis. Spearman rank correlation was used to assess associations between KLRB1 mRNA abundance and each pathway score.

### Predictive value for immunotherapy response

The Tumor Immune Dysfunction and Exclusion (TIDE) framework and SubMap were used only as computational, hypothesis-generating estimates of potential immune-checkpoint-blockade sensitivity. IMvigor210, an anti-PD-L1-treated metastatic urothelial carcinoma cohort, served as a non-DLBCL external comparison. These analyses were not considered sufficient to establish KLRB1/CD161 as a predictive biomarker in DLBCL.

### Drug sensitivity prediction

Pharmacogenomic inference of drug responsiveness was conducted using CTRP and PRISM. The pRRophetic ridge-regression framework was used to estimate area under the dose-response curve (AUC) values from bulk expression profiles. Compounds with differential predicted AUC values between KLRB1-high and KLRB1-low groups were prioritized as exploratory candidates. These *in silico* estimates were not interpreted as clinical drug-response measurements.

### Genomic and epigenetic analysis

Chromosomal arm-level and focal somatic copy-number alterations were catalogued using GISTIC2 on TCGA SNP-array data, and the mutational landscape was visualized with maftools. Illumina HumanMethylation450 data for TCGA-DLBC were obtained from UCSC Xena. CpG sites differentially methylated between the KLRB1-high and KLRB1-low groups were identified using limma and annotated against the hg19 reference genome.

### Single-cell RNA sequencing analysis

Single-cell transcriptomic data from two DLBCL lymph-node biopsies were obtained from the heiDATA repository. Quality control retained cells with 200–7,500 detected genes and <15% mitochondrial reads. Normalization, scaling, dimensionality reduction, and clustering were performed in Seurat (version 4.4.0), with Harmony used for sample integration. Cell identities were assigned using SingleR and canonical lineage markers. Given the two-biopsy sample size and the absence of a dedicated malignant-cell inference workflow, the single-cell analyses were considered exploratory and were not used to make definitive cell-of-origin or malignant B-cell-specific claims. With only two biopsies, residual patient-specific and batch-related effects cannot be reliably separated from biological variation even after Harmony integration.

### High-dimensional weighted gene co-expression network analysis

To identify single-cell co-expression programs associated with KLRB1 mRNA, hdWGCNA was applied to the preprocessed scRNA-seq dataset. The network included genes detected in at least 5% of cells and used a soft-thresholding power of 9. Modules correlated with KLRB1 abundance were identified, and genes in the module of greatest interest were subjected to KEGG pathway overrepresentation analysis. Module-pathway results were used only to prioritize hypotheses and were not interpreted as reproducible disease-wide co-expression networks.

### Pseudotime trajectory and cell–cell communication analysis

T lymphocytes were extracted from the global scRNA-seq dataset and independently re-clustered. Developmental ordering of T-cell transcriptional states was reconstructed through pseudotime trajectory inference with Monocle2. Cell–cell communication networks were probed via the iTALK R toolkit, which detects candidate ligand–receptor pairings spanning functional groupings such as cytokines, growth factors, and immune checkpoint molecules. Pseudotime was treated as a descriptive ordering of transcriptional states, whereas iTALK outputs were treated only as candidate ligand-receptor pairs; neither analysis was considered evidence of lineage progression or active communication.

### Cell lines, culture, and stable transfection

DLBCL cell lines representing the ABC (OCI-LY3 and SU-DHL-2) and GCB (SU-DHL-4 and OCI-LY7) subtypes were cultured in RPMI-1640 with 10% fetal bovine serum and 1% penicillin-streptomycin. Cryopreserved human peripheral-blood CD3^+^ pan T cells were purchased from Lonza (Cat. No. 2W-350) and maintained in RPMI-1640 containing 5% human AB serum, 1% penicillin-streptomycin, and recombinant IL-2 (50 U/mL). OCI-LY3 was selected for genetic manipulation because it represents the ABC subtype and showed comparatively high surface CD161 expression. Stable CD161-overexpressing (OE-CD161) and CD161-knockdown (sh-CD161) OCI-LY3 cells were generated by lentiviral transduction (MOI 20) followed by puromycin selection, with empty-vector controls prepared in parallel and surface expression verified by flow cytometry.

### Flow cytometry

For surface immunophenotyping, cells were stained with fluorochrome-conjugated antibodies against CD161, CD3, CD69, PD-1, and CTLA-4, together with matched isotype controls, for 30 min at 4 °C. For T-cell activation assays, genetically modified DLBCL cells and peripheral-blood-derived CD3^+^ T cells were co-cultured in 0.4-µm Transwell inserts for 48 h, and CD3^+^CD69^+^ T cells in the lower compartment were quantified. For exploratory fixed-dose apoptosis assays, cells were treated with dasatinib (10 μmol/L), doxorubicin (1 μmol/L), or vehicle for 48 h before Annexin V-FITC/PI staining. These experiments were not designed to determine dose-response curves or IC50 values. Data were acquired by flow cytometry and analyzed in FlowJo.

### Western blotting

Whole-cell protein lysates were prepared in RIPA lysis buffer fortified with protease and phosphatase inhibitor cocktails. Equivalent protein quantities were fractionated by SDS-PAGE, electroblotted onto PVDF membranes, and blocked using 5% (w/v) non-fat dry milk. Blots were then probed at 4 °C overnight with primary antibodies recognizing CD161, MHC-I, phospho-mTOR, and GAPDH, after which HRP-linked secondary antibodies were applied. Immunoreactive signals were developed by enhanced chemiluminescence substrate and subjected to densitometric quantification with ImageJ software.

### Enzyme-linked immunosorbent assay

Genetically manipulated DLBCL cells were maintained in serum-deprived culture medium for a 24-h period. Conditioned supernatants were harvested, and the levels of secreted CCL2, IL-8, and TNF-α were quantified by commercial enzyme-linked immunosorbent assay (ELISA) kits in accordance with each manufacturer’s instructions. Absorbance readings were taken at 450 nm, with cytokine concentrations back-calculated from concurrently generated standard curves.

### Statistical analysis

Data processing and statistical analyses were performed in R and GraphPad Prism. Two-group comparisons used the unpaired Student t-test or Wilcoxon rank-sum test according to distributional assumptions. Comparisons involving three or more groups used one-way ANOVA with Fisher’s least significant difference *post hoc* test. Spearman’s rank correlation assessed monotonic relationships, with continuous KLRB1 mRNA retained whenever a correlation was evaluated. A nominal two-sided P < 0.05 was used for prespecified low-dimensional comparisons. A unified Benjamini-Hochberg false-discovery-rate correction was not available across the original high-dimensional correlation, pharmacogenomic, and immunotherapy screens; consequently, nominal P-values from those screens were not treated as confirmatory evidence, and the corresponding findings are reported only as exploratory signals requiring independent validation. Patient-level vital-status comparisons were not treated as formal survival analyses, and no claim of independent prognostic value was made without multivariable Cox regression.

## Results

### Pan-cancer immunological landscape of KLRB1 mRNA

We first mapped pan-cancer immunological correlates of KLRB1 mRNA. Across TCGA tumor types, KLRB1 abundance showed predominantly inverse associations with immunomodulators, CIBERSORT-derived immune-cell estimates, and inhibitory immune-checkpoint transcripts, including CD274 (PD-L1), PDCD1 (PD-1), and CTLA4 (Figures 1A–C). KLRB1 also correlated negatively with TMB and MSI in many malignancies (Supplementary Figures S1A, B). Because these are bulk-tumor associations, their direction may be influenced by cancer-specific cellular composition, immune infiltration, and tumor purity.

**FIGURE 1:**
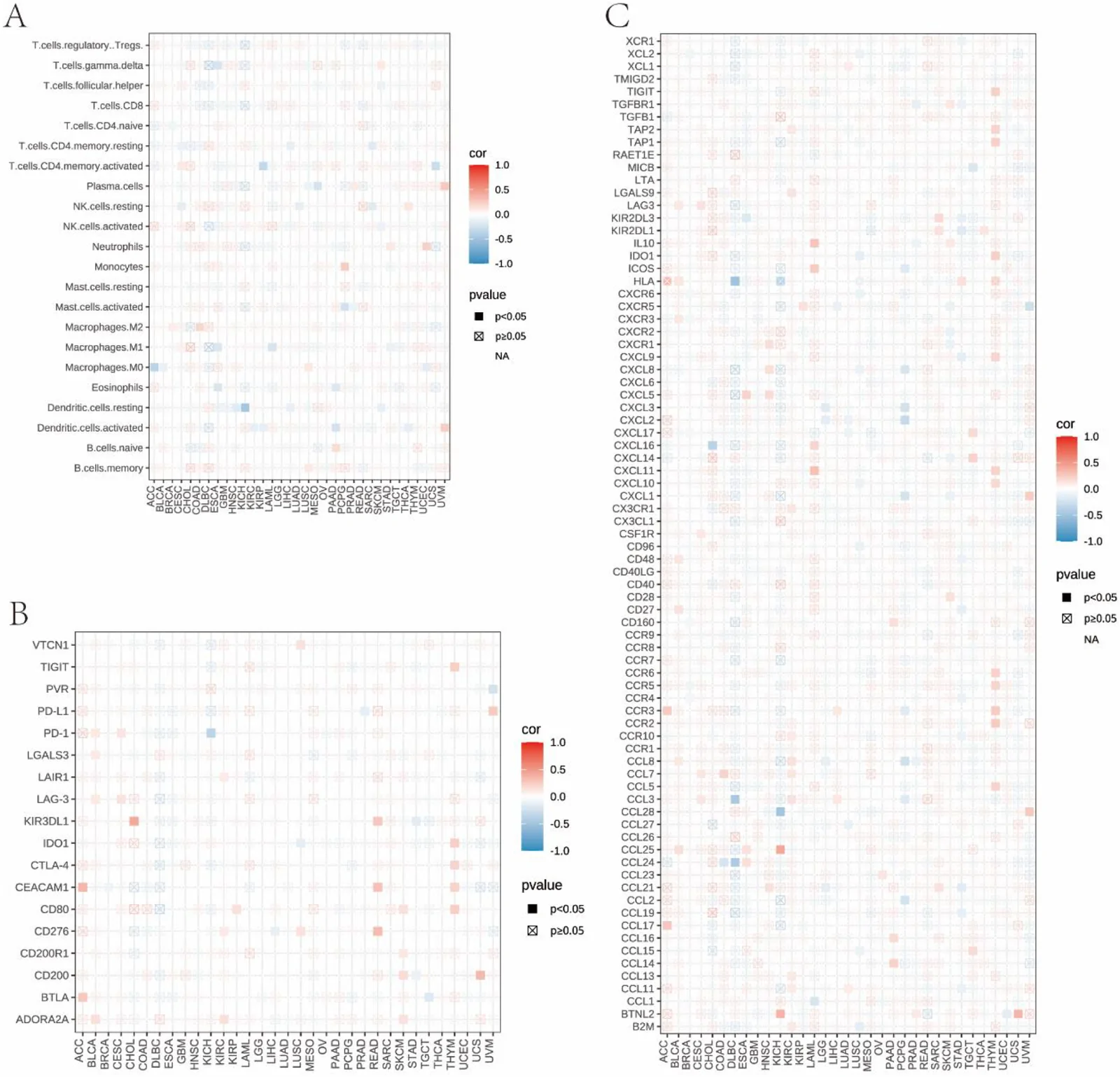
Pan-cancer immunological correlates of KLRB1 mRNA. **(A)** Correlations between KLRB1 transcript abundance and immunomodulatory genes across TCGA tumor types. **(B)** Associations with CIBERSORT-derived immune-cell estimates. **(C)** Correlations with selected inhibitory immune-checkpoint transcripts. These bulk-tumor associations may be influenced by cancer-specific cellular composition and tumor purity.

### KLRB1 mRNA defines an inflamed bulk-tumor phenotype in DLBCL

A descriptive difference from the predominantly inverse pan-cancer pattern was observed in DLBCL bulk-tumor data; however, a formal KLRB1-by-disease interaction model was not performed. Higher KLRB1 mRNA was positively associated with numerous immunomodulators, including MHC class I and II genes (Figure 2A), and with immune-cell infiltration estimates across seven deconvolution methods (Figure 2B). KLRB1 was also positively correlated with PDCD1, CTLA4, LAG3, HAVCR2, and IDO1 (Figure 2C), consistent with an immune-inflamed but potentially exhausted microenvironment. These correlations do not establish expression by malignant B cells: because KLRB1 is expressed by multiple T-, NK-, and some B-cell populations, variation in immune-cell abundance and tumor purity may contribute substantially to the bulk signal. Within DLBCL, KLRB1 remained inversely associated with TMB and MSI (Supplementary Figures S2A, B), supporting a disease-context-dependent association rather than a universal mechanism.

**FIGURE 2:**
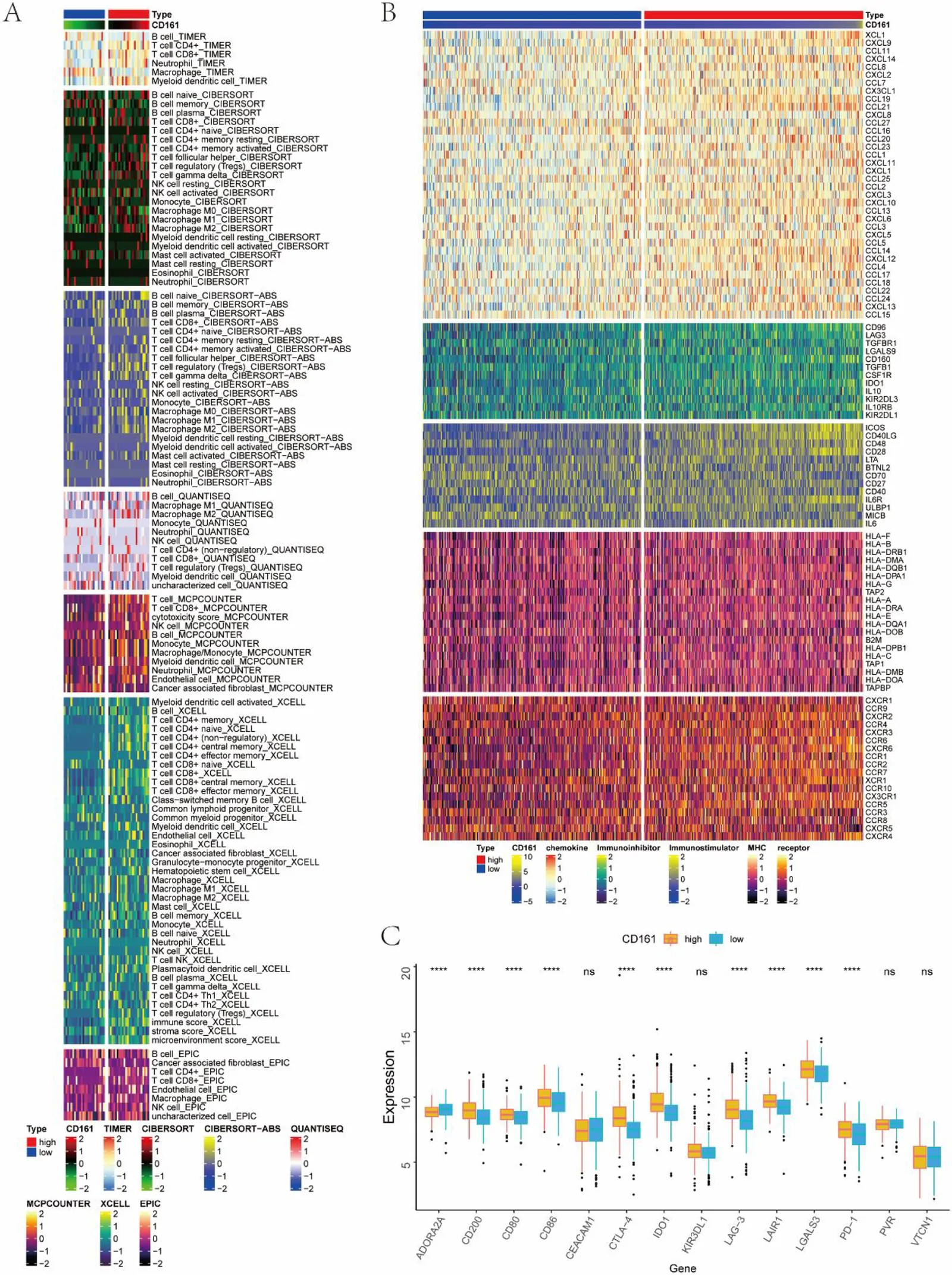
KLRB1 mRNA marks an immune-inflamed bulk-tumor phenotype in DLBCL. **(A)** Correlations with immunomodulators, including MHC class I and II genes. **(B)** Associations with immune-cell infiltration estimates across seven methods. **(C)** Correlations with PDCD1, CTLA4, LAG3, HAVCR2, and IDO1. The analysis does not by itself identify the cellular source of KLRB1.

### Clinical associations and subtype specificity of KLRB1 mRNA in DLBCL

In DLBCL, KLRB1 mRNA abundance was associated with vital status at last follow-up and clinical stage (Figure 3A). KLRB1 was also higher in ABC than in GCB tumors (Figure 3B), and this subtype-related pattern was reproduced in GSE4475 and GSE10846 (Figure 3C). Because the analysis compared cross-sectional vital-status groups rather than time-to-event outcomes and did not include multivariable Cox modeling, these findings support an association with clinical characteristics but do not establish KLRB1/CD161 as an independent prognostic biomarker.

**FIGURE 3:**
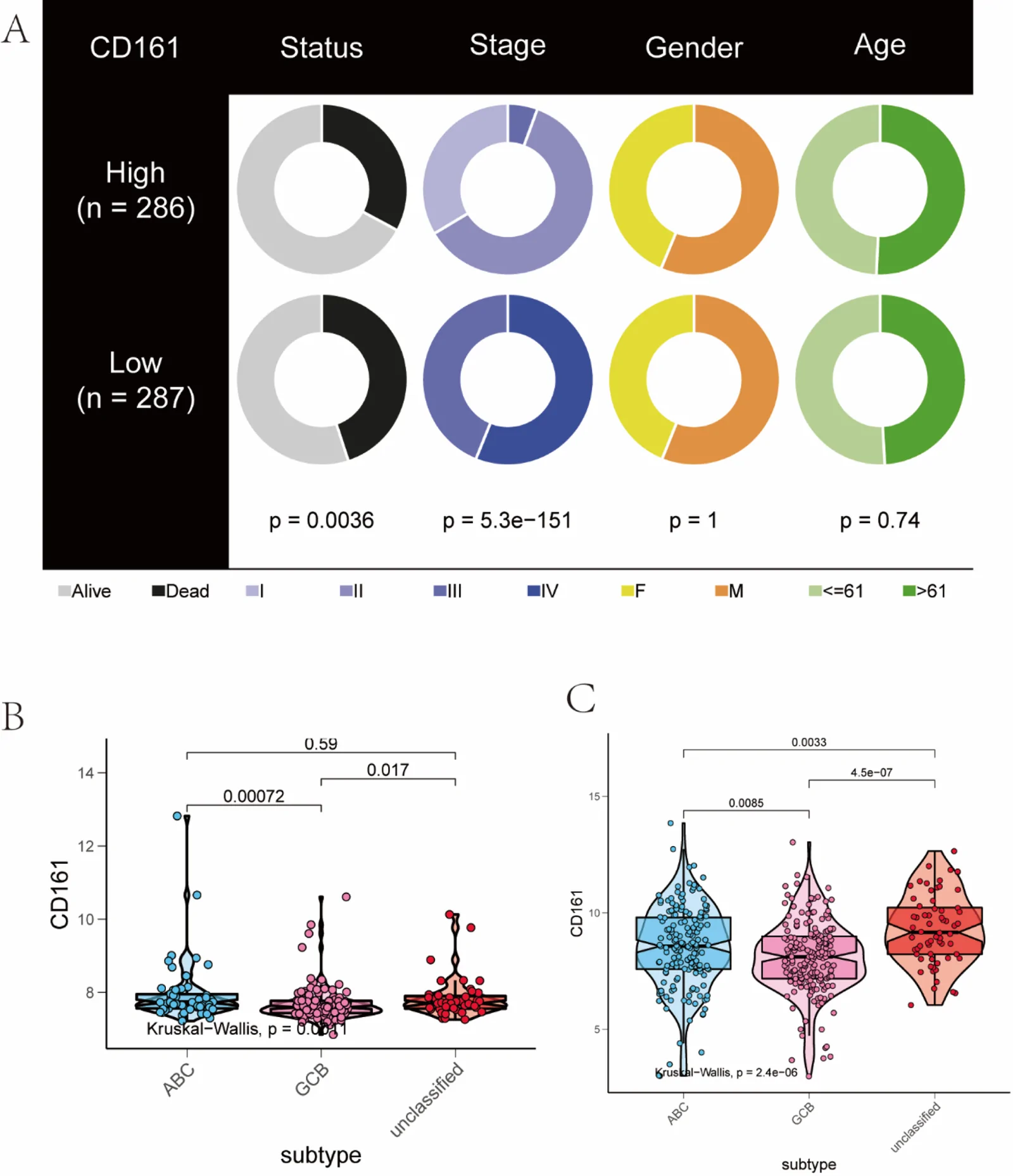
Clinical associations and subtype-dependent KLRB1 mRNA expression in DLBCL. **(A)** Associations with vital status at last follow-up and clinical stage. **(B)** Higher KLRB1 mRNA in ABC than GCB tumors. **(C)** Evaluation of the ABC-versus-GCB pattern in GSE4475 and GSE10846. These comparisons do not establish independent prognostic value.

### Pathway-level correlates of KLRB1 mRNA

To explore pathway-level associations, we examined covariation between KLRB1 mRNA and curated transcriptional signatures. KLRB1 correlated positively with antigen-processing machinery, CD8^+^ T-cell effector, APM_signal, and Proteasome signatures (Figure 4A). In the Hallmark collection, the strongest positive correlation was with allograft rejection and the strongest negative correlation was with MTORC1_SIGNALING (Figure 4B). These transcriptomic relationships are associative and do not by themselves establish a direct signaling pathway from CD161 to mTORC1.

**FIGURE 4:**
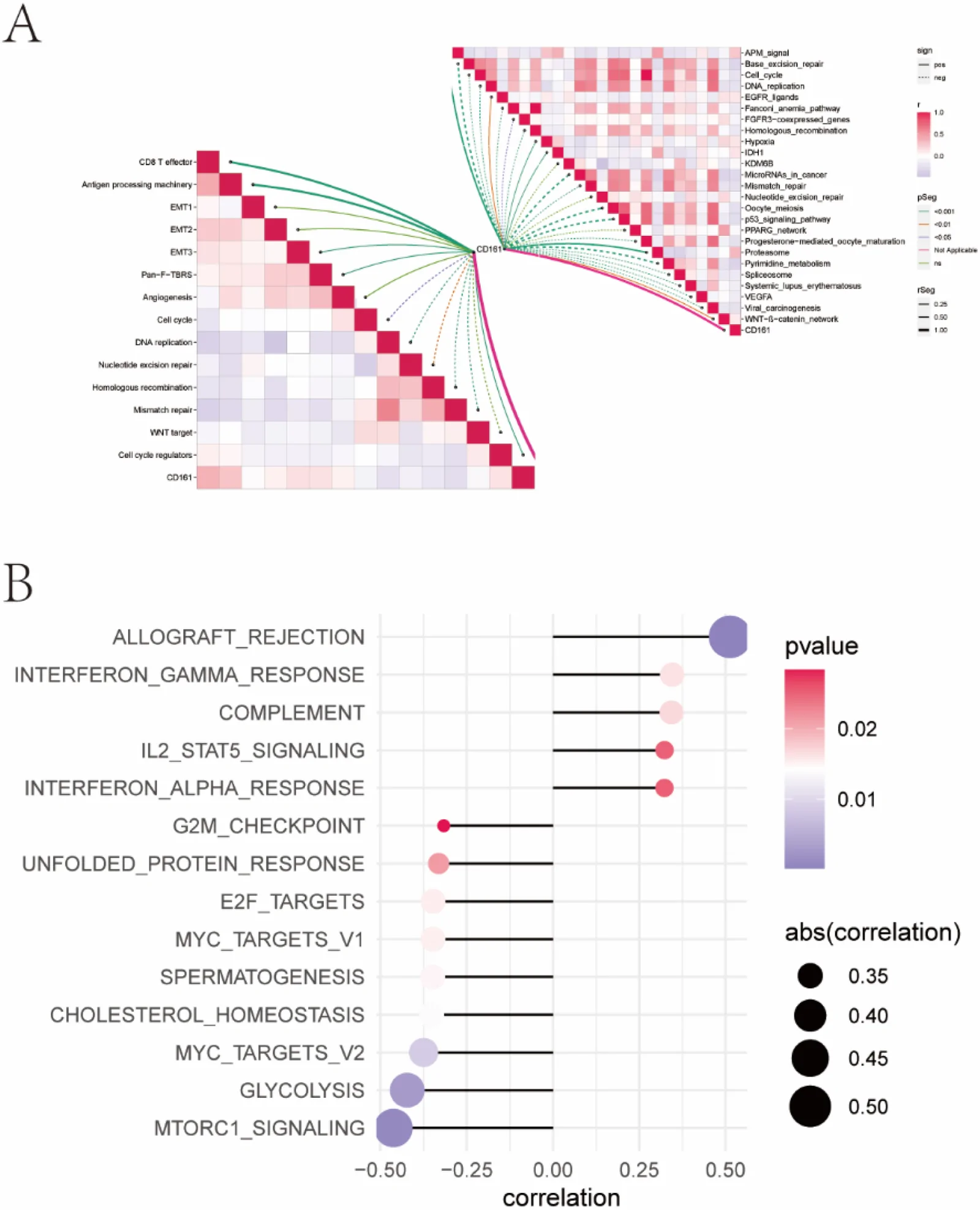
Pathway-level correlates of KLRB1 mRNA in DLBCL. **(A)** Positive correlations with antigen-processing, CD8^+^ T-cell effector, and selected immunotherapy-related signatures. **(B)** Positive association with allograft rejection and inverse association with MTORC1_SIGNALING. Correlations are associative and do not prove direct pathway regulation.

### Exploratory association with computational immunotherapy estimates

We next explored whether KLRB1 mRNA carried hypothesis-generating information related to immune-checkpoint blockade. The KLRB1-high group had lower TIDE scores, and SubMap produced a concordant computational prediction (Figures 5A,B). However, objective-response rates did not differ significantly between KLRB1-high and KLRB1-low groups in IMvigor210, a urothelial carcinoma rather than DLBCL cohort (Figures 5C,D). The negative external result is informative and suggests that any predictive value may be tumor-type specific; it does not establish KLRB1/CD161 as a DLBCL immunotherapy biomarker.

**FIGURE 5:**
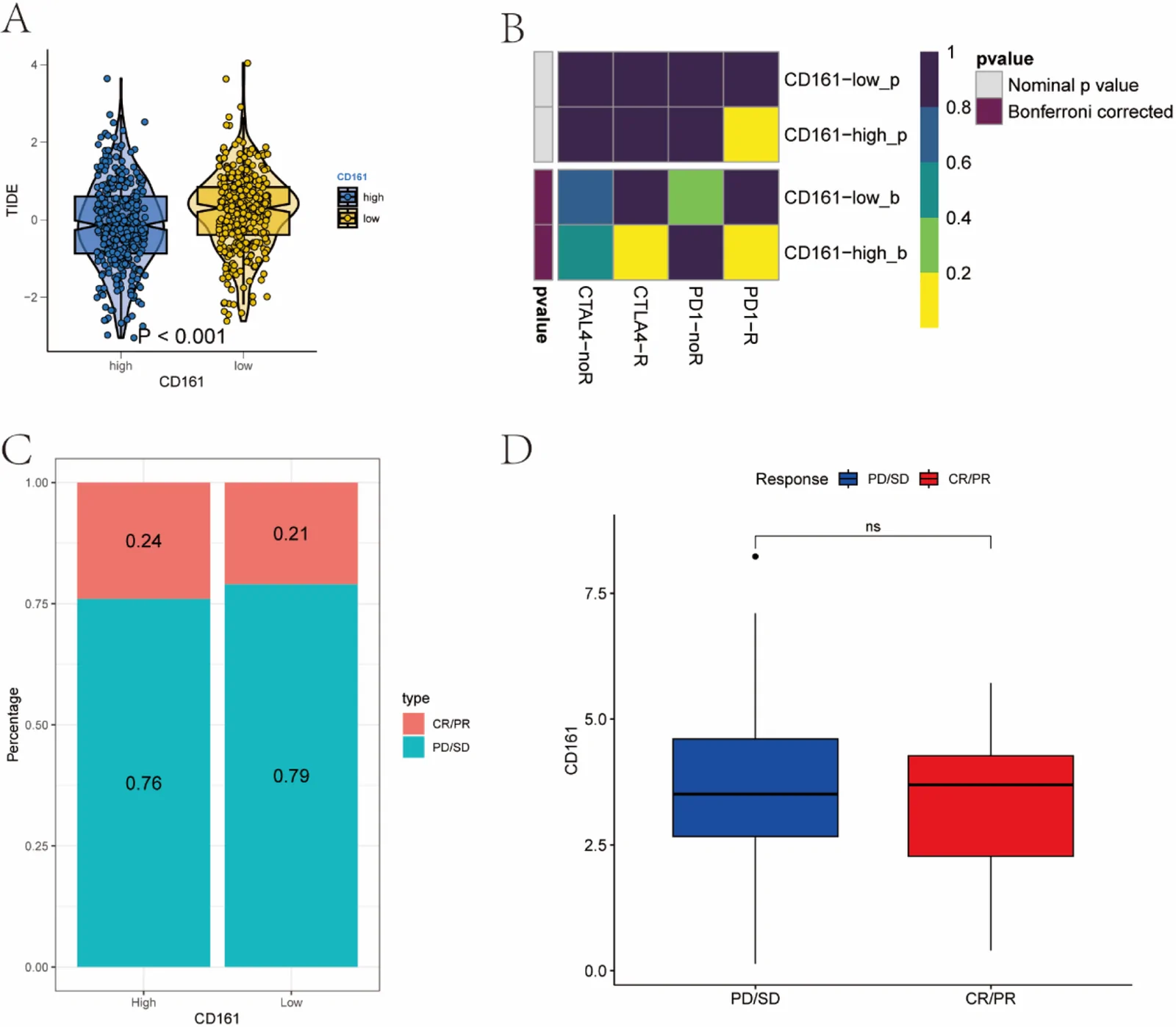
Exploratory computational assessment of KLRB1 mRNA and immunotherapy response. **(A)** TIDE scores by KLRB1 group. **(B)** SubMap prediction. **(C,D)** IMvigor210, an anti-PD-L1-treated urothelial carcinoma cohort, showed no significant objective-response difference. These findings do not establish a DLBCL predictive biomarker.

### Exploratory drug-sensitivity modeling

Integrated CTRP and PRISM modeling suggested lower predicted AUC values for dasatinib, GANT-61, saracatinib, BMS-690514, indirubin, and vandetanib in KLRB1-high DLBCL, whereas predicted doxorubicin AUC was higher (Figures 6A,B). Fixed-dose apoptosis assays in CD161-modified OCI-LY3 cells were directionally consistent for dasatinib and doxorubicin. Because validation used one ABC-subtype cell line, one concentration per drug, and a single exposure duration, these findings should be considered preliminary and do not support clinical treatment selection.

**FIGURE 6:**
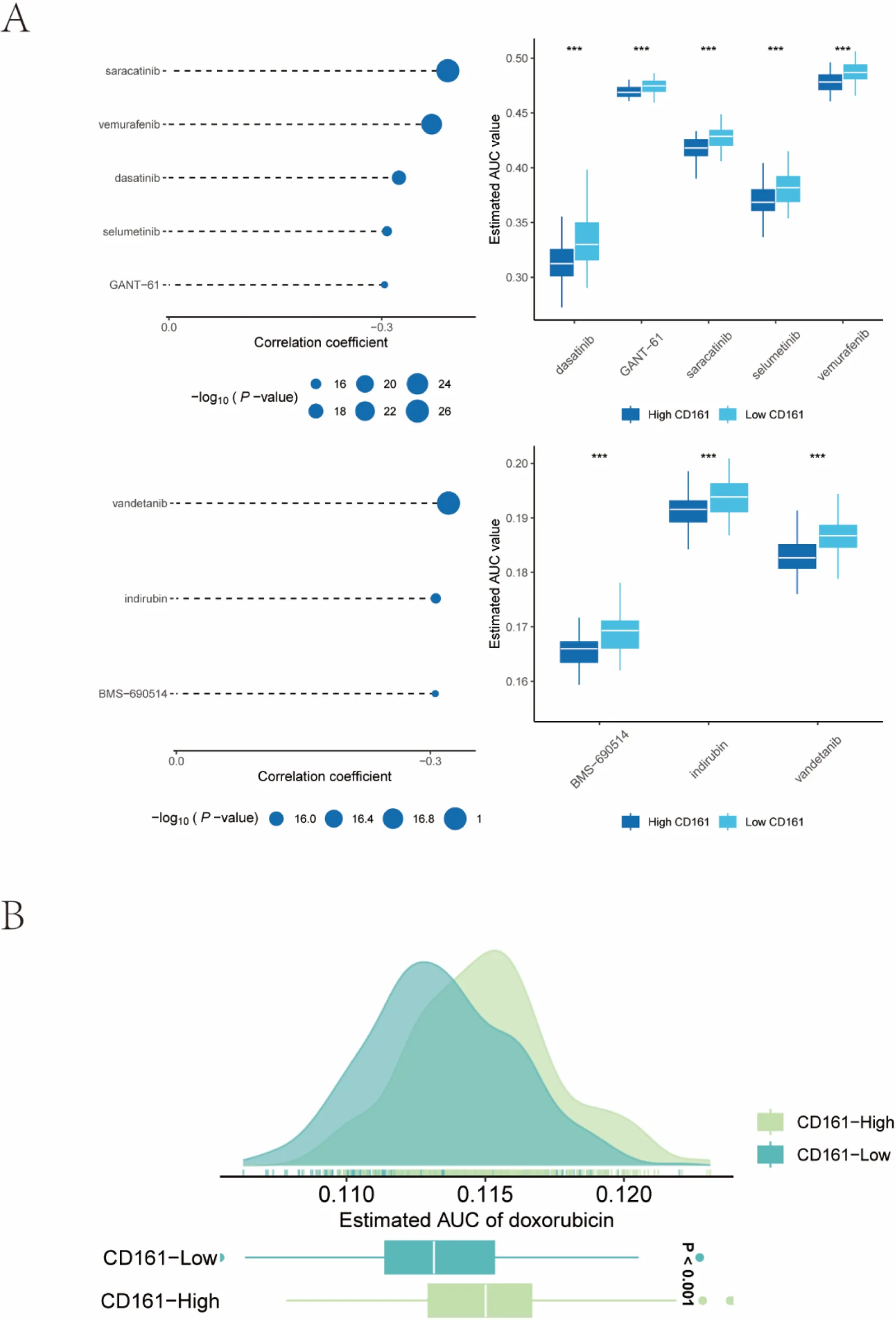
Exploratory pharmacogenomic inference stratified by KLRB1 mRNA. **(A)** Differential predicted AUC values for candidate agents in CTRP and PRISM. **(B)** Higher predicted doxorubicin AUC in the KLRB1-high group. Predictions require dose-response validation in multiple DLBCL models.

### Genomic and epigenetic landscapes of KLRB1-High DLBCL

The KLRB1-low group showed greater burdens of arm-level and focal somatic copy-number alterations and generally higher mutation frequencies among recurrently altered genes (Figures 7A–D). Five CpG sites-cg01260615, cg03967798, cg24122407, cg24529237, and cg25139084-differed between KLRB1-high and KLRB1-low tumors (Figures 7E–I). Array annotation linked cg24122407 to IGF1R and placed cg25139084 near SNIP1, whereas cg03967798 and cg24529237 were intergenic/open-sea loci and cg01260615 lacked a confident gene assignment. These annotations suggest potentially relevant growth-factor and immune-regulatory connections, but locus-specific regulation and causality require experimental validation.

**FIGURE 7:**
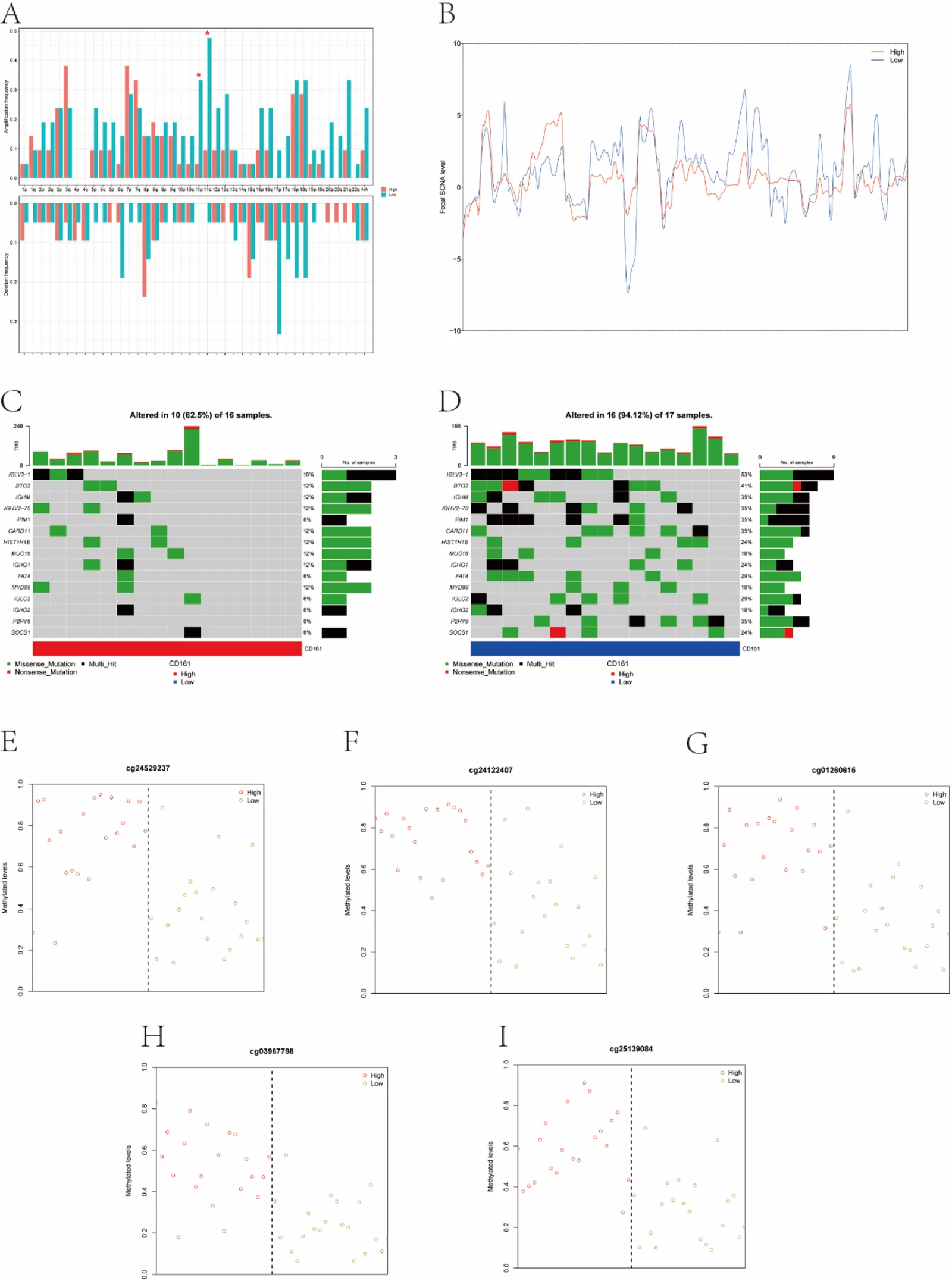
Genomic and epigenomic differences between KLRB1-high and KLRB1-low DLBCL. **(A,B)** Arm-level and focal copy-number alterations. **(C,D)** Recurrently mutated genes. **(E–I)** Differential methylation at cg01260615, cg03967798, cg24122407 (IGF1R), cg24529237, and cg25139084 (near SNIP1). Intergenic/open-sea loci and unassigned probes require functional annotation and validation.

### Single-cell transcriptomics identifies KLRB1-associated co-expression modules

We used scRNA-seq data from two DLBCL lymph-node biopsies to explore the cellular context of KLRB1. Clustering identified T-cell, B-cell, and monocytic populations (Figures 8A,B), and hdWGCNA identified 29 modules, with the green module showing the strongest positive association with KLRB1 mRNA (Figures 8C–F). Green-module genes were enriched for endocytosis and cancer-related pathways (Figure 8G). Owing to the small sample size, absence of robust malignant B-cell inference, and limited representation of rarer lineages such as NK cells, these results are exploratory and should not be generalized as definitive cell-type-specific expression patterns. Patient-level replication was not possible, and no module or enrichment result was considered independently validated.

**FIGURE 8:**
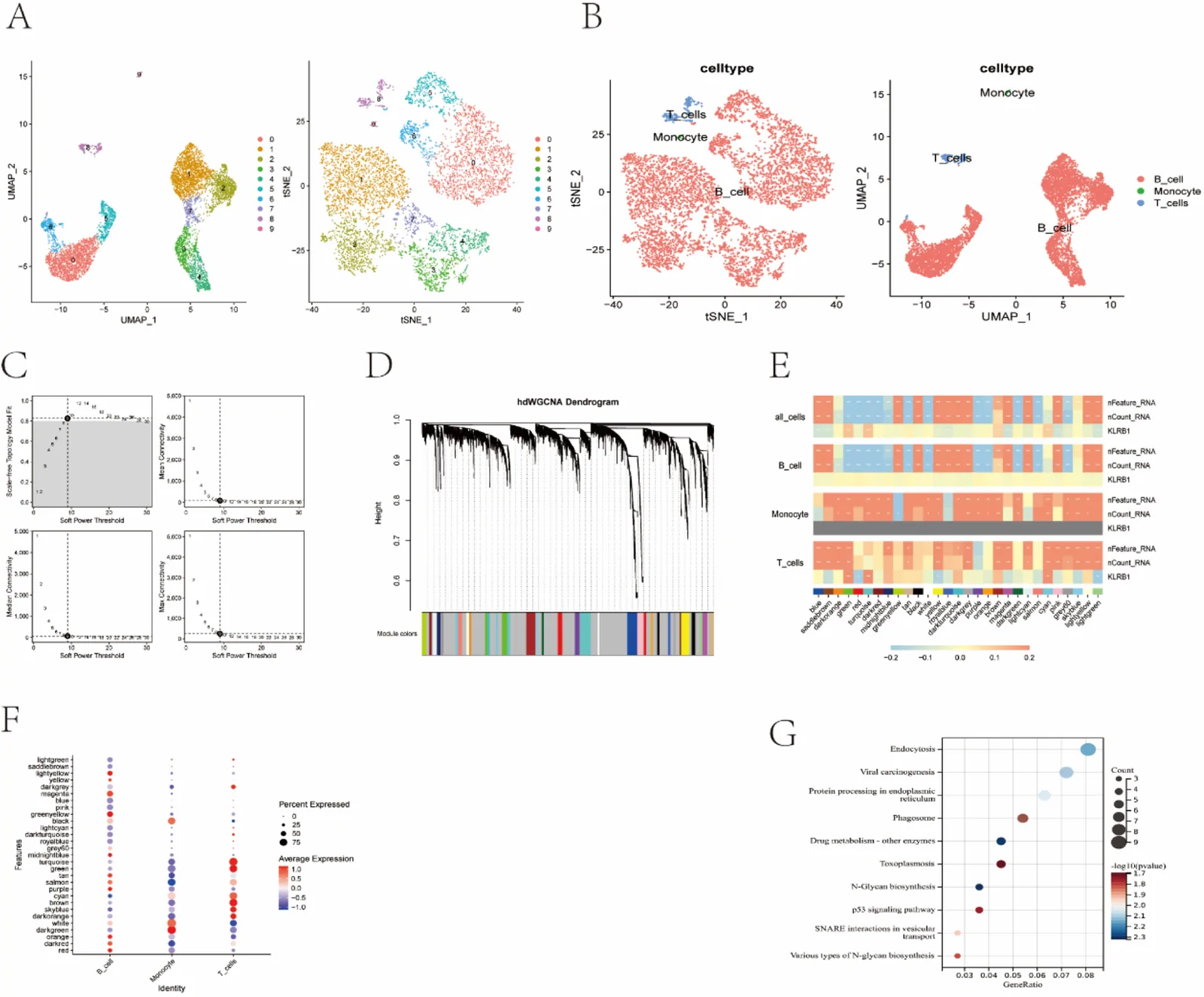
Exploratory single-cell analysis of KLRB1-associated programs in two DLBCL biopsies. **(A,B)** UMAP and broad lineage annotation. **(C–F)** hdWGCNA modules and association of the green module with KLRB1 mRNA. **(G)** KEGG enrichment. The limited cohort and absence of robust malignant-cell inference constrain generalizability.

### T-Cell dynamics and intercellular communication

Further sub-clustering of the T-cell compartment identified three transcriptional states annotated as CD4^+^ central memory, CD4^+^ effector memory, and gamma-delta T cells (Figures 9A,B). Monocle2 generated a putative pseudotime structure among these states (Figures 9C,D), and iTALK identified candidate cytokine, growth-factor, and immune-checkpoint ligand-receptor pairs across annotated cell types (Supplementary Figures S3A–C). Given the two-biopsy cohort and inference-based nature of these methods, the results are exploratory and do not establish lineage relationships, active cell-cell communication, or KLRB1-dependent signaling. Accordingly, these panels should be read as visualizations of testable hypotheses, not as evidence of a biological trajectory or operative signaling circuit.

**FIGURE 9:**
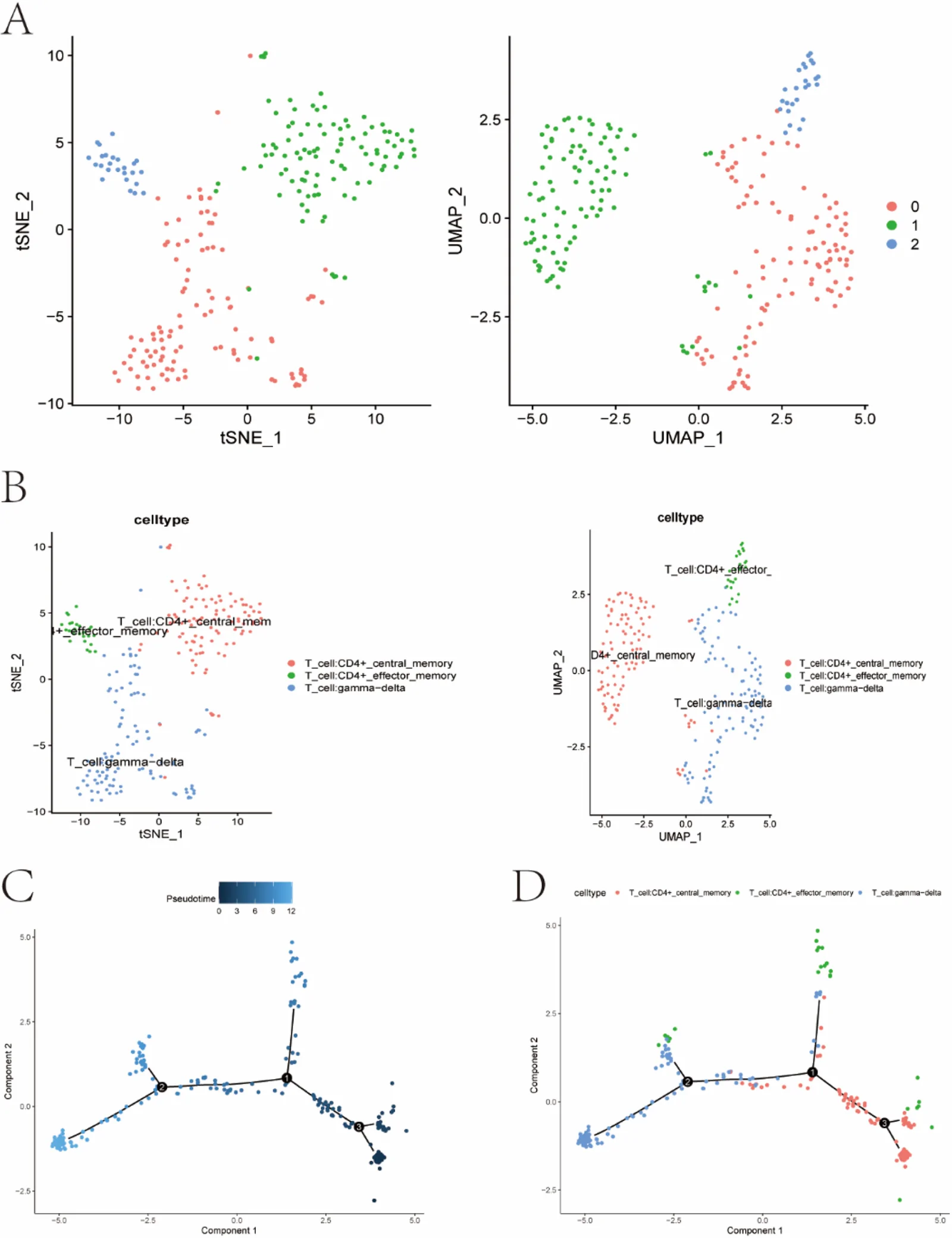
Exploratory T-cell transcriptional states and inferred intercellular communication. **(A,B)** Re-clustering of the T-cell fraction identified CD4^+^ central-memory, CD4^+^ effector-memory, and gamma-delta T-cell states. **(C,D)** Monocle2 generated a putative pseudotime structure. iTALK analysis (Supplementary Figures S3A–C) identified candidate ligand-receptor pairs. These inference-based results from two biopsies do not establish lineage trajectories or active communication.

### Experimental assessment of CD161 protein in DLBCL cells

*In vitro* assays were used to examine the encoded CD161 protein. Flow cytometry showed higher surface CD161 in ABC-type OCI-LY3 and SU-DHL-2 cells than in GCB-type SU-DHL-4 and OCI-LY7 cells (Figure 10A). CD161 overexpression or knockdown in OCI-LY3 altered PD-1 and CTLA-4 surface staining, CD3^+^ T-cell CD69 induction in Transwell co-culture, MHC-I protein abundance, phospho-mTOR, cytokine secretion, and fixed-dose drug-induced apoptosis (Figures 10B–F). These findings support a cell-line-specific functional association but do not establish the full signaling pathway, generalizability across DLBCL subtypes, or clinical drug sensitivity. The absence of functional replication in a GCB line and the lack of drug dose-response curves preclude subtype-wide or pharmacologic conclusions.

**FIGURE 10:**
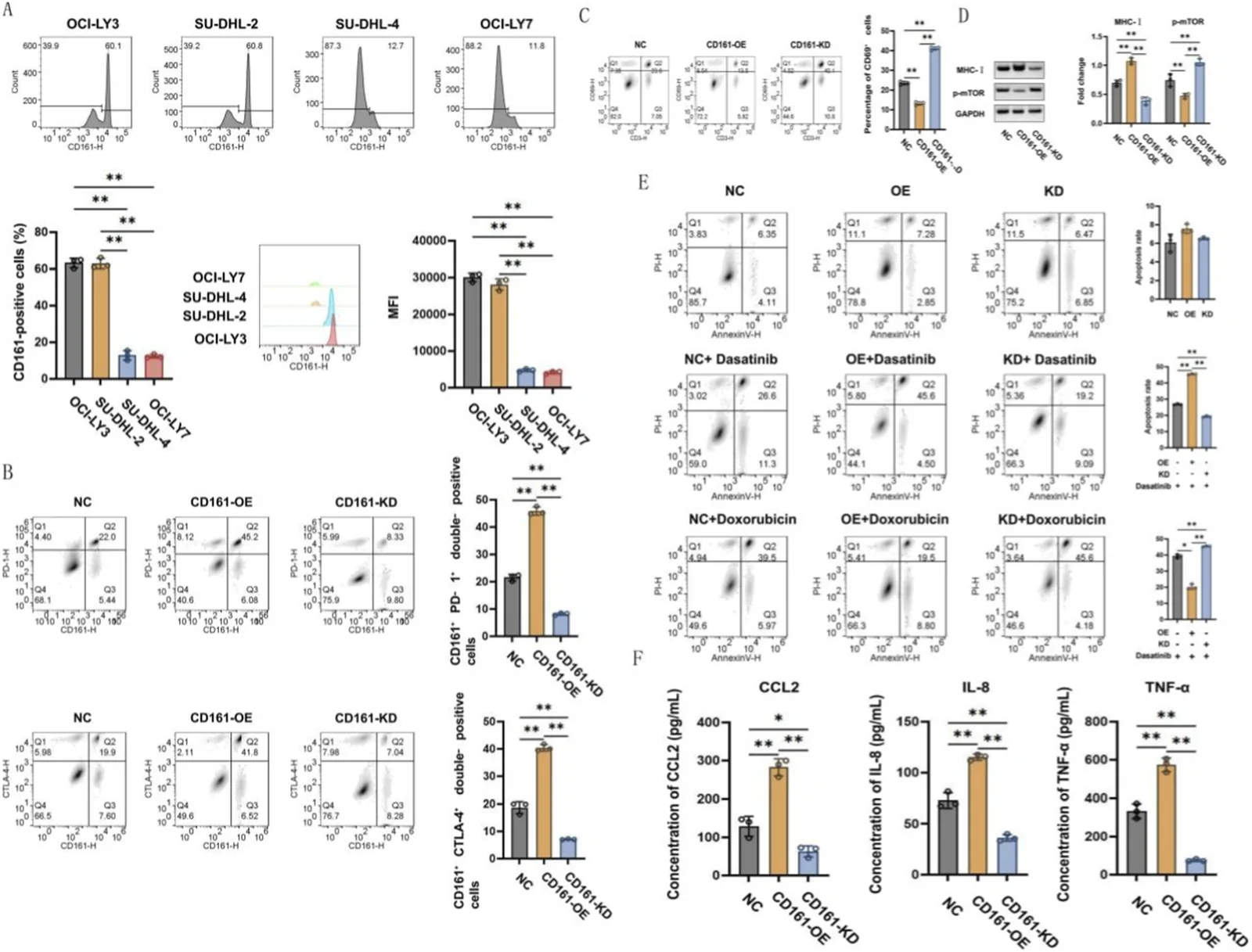
*In vitro* functional assessment of CD161 protein in DLBCL cells. **(A)** Surface CD161 across ABC and GCB cell lines. **(B–D)** Effects of CD161 manipulation in OCI-LY3 on immune-checkpoint staining, T-cell CD69 induction, MHC-I, and phospho-mTOR. **(E)** Fixed-dose dasatinib- and doxorubicin-induced apoptosis. **(F)** Cytokine secretion. Findings are limited to the stated cell-line and assay conditions.

## Discussion

The genetic, epigenetic, and phenotypic diversity of DLBCL limits the precision of existing clinicopathological classifications (Pasqualucci and Dalla-Favera, 2018; Sarode et al., 2025). CAR-T outcomes in lymphoma can also be influenced by patient comorbidities, including renal dysfunction (Ahmed et al., 2022), while the expanding range of agents and regimens reflects the need for individualized treatment (Wang et al., 2020). Its heterogeneous immune microenvironment contains effector populations with context-dependent functions: CD8^+^ T cells can eliminate immunogenic malignant clones, whereas sustained immune pressure and associated metabolic adaptation may contribute to disease progression (Park et al., 2023; Ringel et al., 2020). Against this background, the present integrative analyses nominate the KLRB1 mRNA/CD161 protein axis as an immune-associated candidate for further mechanistic study, rather than as an established nodal regulator or clinical biomarker. Contemporary synthesis of DLBCL biology likewise places genetic lesions, altered signaling, immune composition, and emerging targeted therapies within an integrated disease framework (Sarode et al., 2025).

Our pan-cancer survey found predominantly inverse associations between bulk-tumor KLRB1 mRNA and immune features, whereas DLBCL showed a positive, immune-inflamed pattern. Several complementary explanations are plausible. First, bulk KLRB1 may chiefly reflect differences in T-, NK-, and other immune-cell infiltration and tumor purity rather than malignant B-cell expression. Second, the DLBCL microenvironment, in which CLEC2D/LLT1 may be expressed by malignant and myeloid populations, could alter the functional context of CD161 signaling; however, this possibility was not directly tested here. CD161 expression has also been documented in a subset of T-cell prolymphocytic leukemia cases (Gilles et al., 2019). Third, DLBCL-specific transcription-factor networks, chronic NF-kB activity, and epigenetic remodeling could rewire downstream responses or co-regulate KLRB1 with antigen-presentation and exhaustion programs. These hypotheses are testable through cell-type-resolved KLRB1/CLEC2D profiling, tumor-purity-adjusted models, chromatin-accessibility analysis, and perturbation studies in matched ABC and GCB systems.

Although published work supports biological relevance of the CD161-CLEC2D/LLT1 axis in hematologic malignancies (Alvarez et al., 2024), the present study did not perform a direct cohort-level analysis of CLEC2D expression, subtype differences, clinical outcomes, or correlation with KLRB1. Consequently, CLEC2D is discussed as a literature-supported mechanistic context rather than as a result established by the current dataset. A joint, cell-type-resolved analysis of receptor and ligand expression is a priority for future work. This omission also means that the current manuscript cannot determine whether cohort-level CLEC2D abundance modifies the association between KLRB1 and immune features.

The inverse associations of KLRB1 mRNA with TMB and MSI in DLBCL warrant cautious interpretation. A previous pan-cancer study likewise reported an inverse correlation between KLRB1 transcript abundance and MSI across several malignancies (Cheng et al., 2022). However, correlation with these genomic features does not demonstrate predictive value for immunotherapy or prognosis. Future analyses should adjust for molecular subtype, tumor purity, and other clinical covariates and test whether KLRB1 adds information beyond established genomic and immune markers.

Prior studies have linked tumor mutational burden and single-cell immune ecosystems to prognosis and tumor biology (Lee et al., 2019; Sun et al., 2021), while a pan-cancer analysis specifically associated CD161 with patient prognosis (Ye et al., 2021). In the present DLBCL cohorts, KLRB1 mRNA differed by vital status at last follow-up and clinical stage and was consistently higher in ABC than in GCB tumors. These observations may indicate a compensatory immune-engagement program in ABC DLBCL. However, cross-sectional vital-status comparisons are not equivalent to Kaplan-Meier or Cox analyses, and the present data do not establish KLRB1/CD161 as an independent prognostic biomarker after adjustment for age, sex, stage, molecular subtype, or other clinical covariate.

CD161 expression has been reported to define rapidly responding effector CD4^+^ T cells associated with improved survival in HPV16-associated tumors (Duurland et al., 2022). KLRB1 mRNA correlated with antigen-processing machinery and CD8^+^ T-cell effector programs; the integrity of antigen processing and presentation is relevant to immune-checkpoint inhibitor efficacy (Mpakali and Stratikos, 2021). CD161 has also been proposed as a potential immune checkpoint in pan-cancer analyses (Zhou et al., 2021). Nevertheless, the lower TIDE score in the KLRB1-high group remains a computational observation rather than evidence of clinical benefit from immune-checkpoint blockade. The inverse association with MTORC1_SIGNALING and the reduction in phospho-mTOR after CD161 overexpression raise a mechanistic hypothesis. Possible intermediate nodes include PI3K-AKT signaling, AMPK-dependent energy sensing, and amino-acid-sensing inputs to the Ragulator-Rag-mTORC1 axis (Szwed et al., 2021; Popova and Jücker, 2021). Whether CD161 engagement directly affects these nodes, or instead acts indirectly through changes in cellular state or cytokine signaling, should be tested with time-resolved phosphorylation assays, pathway inhibitors, and rescue experiments.

Pharmacogenomic modeling suggested that KLRB1-high tumors may differ in sensitivity to dasatinib and doxorubicin, and fixed-dose apoptosis assays in CD161-modified OCI-LY3 cells showed directionally consistent effects. These data are preliminary: they do not define IC50 values, clinically achievable exposure-response relationships, or generalizability beyond one ABC-subtype cell line. Validation in additional ABC and GCB models, dose-response experiments, and pharmacokinetic-aware *in vivo* studies is required before considering KLRB1/CD161 for treatment selection. More broadly, computer-aided drug design and pharmacophore-based virtual-screening studies illustrate how *in silico* approaches can prioritize candidate compounds, but their predictions require experimental validation (Yang et al., 2018).

Genomically, the KLRB1-low subset showed a higher burden of copy-number and somatic alterations. Annotation of the five differential CpG sites added biological context: cg24122407 maps to IGF1R, a growth and survival receptor with established relevance to oncogenic signaling; cg25139084 lies near SNIP1, a regulator connected to NF-kB/TGF-beta transcriptional control; cg03967798 and cg24529237 are intergenic/open-sea loci; and cg01260615 lacks a confident gene annotation. These loci may mark broader epigenetic states rather than directly control KLRB1, and targeted methylation-expression analyses are needed. The two-biopsy hdWGCNA, pseudotime, and cell-communication findings should likewise be regarded as exploratory. More generally, alterations in DNA damage-response networks can shape genomic instability and therapeutic vulnerability across malignancies (Zhu et al., 2026), while experimental suppression of the homologous-recombination factor XRCC2 has increased sensitivity to DNA-damaging treatment in preclinical tumor models (Wang et al., 2014). Structure-based virtual screening has also been used to identify putative PTP-LAR inhibitors (Du et al., 2020), further illustrating that computationally prioritized candidates require experimental confirmation. Importantly, coding mutations and copy-number changes represent only part of the lymphoma genomic landscape. Noncoding mutations can retarget super-enhancers; at the PAX5/ZCCHC7 locus, this process has been linked to altered rRNA processing and protein-synthesis dysregulation during progression from follicular lymphoma to high-grade B-cell lymphoma (Leeman-Neill et al., 2023). Although this mechanism was not tested here, it underscores why regulatory noncoding alterations should be incorporated into future KLRB1-associated genomic analyses.

Several limitations should be considered. First, the scRNA-seq analysis comprised only two lymph-node biopsies. Patient variation and residual batch effects therefore cannot be disentangled reliably, malignant from non-malignant B cells were not robustly distinguished, and the hdWGCNA, pseudotime, and cell-cell communication outputs are strictly hypothesis-generating. Independent validation in larger datasets such as GSE182434 and GSE242756, as well as spatial transcriptomic cohorts, is needed. Second, bulk KLRB1 mRNA is sensitive to immune-cell composition and tumor purity, and a direct cohort-level CLEC2D analysis was not performed; future analyses should use purity-adjusted, cell-type-specific, and joint KLRB1-CLEC2D models. Third, the descriptive difference between DLBCL and the pan-cancer association pattern was not tested with a KLRB1-by-disease interaction term. Fourth, no unified false-discovery-rate correction was available across the original high-dimensional screens; those findings must therefore remain exploratory until independently replicated with adjusted P-values. Fifth, median-based group comparisons were not tested across alternative cutoffs, so no clinically transferable KLRB1 threshold can be inferred; continuous-expression analyses should be primary in future validation. Sixth, TIDE and SubMap are computational predictions, whereas IMvigor210 is a urothelial carcinoma cohort with a negative response comparison; validation is required in DLBCL-specific immunotherapy cohorts. Seventh, functional and drug validation was limited to OCI-LY3, fixed concentrations, and a 48-h exposure, without GCB-line replication, dose-response curves, or IC50 estimation. Finally, the clinical analysis did not establish independent prognostic value through multivariable Cox regression, and all cell-based findings require confirmation in additional ABC and GCB models and *in vivo*.

## Conclusion

In summary, this multi-omics study identifies an association between the KLRB1 mRNA/CD161 protein axis and an immune-inflamed DLBCL phenotype, ABC subtype biology, antigen-presentation programs, and reduced mTORC1-related signaling. Computational immunotherapy and drug-response findings, single-cell analyses, and OCI-LY3 experiments provide convergent but preliminary evidence. KLRB1/CD161 should therefore be viewed as a hypothesis-generating immune-associated candidate rather than an established independent prognostic biomarker, treatment predictor, or validated therapeutic target in DLBCL. In particular, the present data do not establish a validated KLRB1 threshold, a DLBCL-specific disease interaction, a CLEC2D-dependent mechanism, reproducible single-cell trajectories, or subtype-generalizable drug sensitivity.

## Data Availability

The original contributions presented in the study are included in the article/Supplementary Material, further inquiries can be directed to the corresponding authors.
