# Supplementary material for: Integrative multi-omics characterization of KLRB1/CD161 in diffuse large B-cell lymphoma: associations with immune context, subtype biology, and therapeutic response
Source: Front Mol Biosci. 2026 Sep 24;13:1903725. doi: 10.3389/fmolb.2026.1903725 (PMC13645582; doi:10.3389/fmolb.2026.1903725)
Supplement: Supplementary file 1 [file Supplementaryfile1.docx]

**Figure S1. Pan-cancer association of CD161 with tumor mutational burden and microsatellite instability.
(A) Correlation of CD161 expression with tumor mutational burden (TMB) across various cancer types. (B) Correlation of CD161 with microsatellite instability (MSI) in pan-cancer analysis. CD161 was inversely associated with both TMB and MSI in most cancers.**

**Figure S2. CD161 association with TMB and MSI specifically in DLBC.
(A) CD161 expression negatively correlated with TMB in DLBC. (B) CD161 expression negatively correlated with MSI in DLBC, contrasting with the pan-cancer trend.**

**Figure S3. Cell-cell communication network analysis by iTALK in the DLBC tumor microenvironment.**

(A) Global ligand-receptor interaction network among the identified cell populations (T cells, B cells, monocytes) in DLBC scRNA-seq data. (B) Cytokine-focused subnetwork showing predominant ligand-receptor pairs involved in inflammatory and immune regulation. (C) Subnetwork highlighting interactions mediated by growth factors and immune checkpoints. The analysis reveals a complex intercellular communication landscape, with CD161-associated cells engaging in extensive crosstalk through diverse signaling axes.
